# A Single Biosynthetic Gene Cluster Is Responsible for the Production of Bagremycin Antibiotics and Ferroverdin Iron Chelators

**DOI:** 10.1128/mBio.01230-19

**Published:** 2019-08-13

**Authors:** Loïc Martinet, Aymeric Naômé, Benoit Deflandre, Marta Maciejewska, Déborah Tellatin, Elodie Tenconi, Nicolas Smargiasso, Edwin de Pauw, Gilles P. van Wezel, Sébastien Rigali

**Affiliations:** a InBioS—Centre for Protein Engineering, Institut de Chimie B6a, University of Liège, Liège, Belgium; b MolSys Research Unit, Mass Spectrometry Laboratory, University of Liège, Liège, Belgium; c Molecular Biotechnology, Institute of Biology, Leiden University, Leiden, The Netherlands; McMaster University

**Keywords:** *Streptomyces*, genome analysis, iron regulation, natural antimicrobial products, secondary metabolism

## Abstract

Biosynthetic gene clusters (BGCs) are organized groups of genes involved in the production of specialized metabolites. Typically, one BGC is responsible for the production of one or several similar compounds with bioactivities that usually only vary in terms of strength and/or specificity. Here we show that the previously described ferroverdins and bagremycins, which are families of metabolites with different bioactivities, are produced from the same BGC, whereby the fate of the biosynthetic pathway depends on iron availability. Under conditions of iron depletion, the monomeric bagremycins are formed, representing amino-aromatic antibiotics resulting from the condensation of 3-amino-4-hydroxybenzoic acid with *p*-vinylphenol. Conversely, when iron is abundantly available, the biosynthetic pathway additionally produces a molecule based on *p*-vinylphenyl-3-nitroso-4-hydroxybenzoate, which complexes iron to form the trimeric ferroverdins that have anticholesterol activity. Thus, our work shows a unique exception to the concept that BGCs should only produce a single family of molecules with one type of bioactivity and that in fact different bioactive molecules may be produced depending on the environmental conditions.

## Abstract (precis)

Access to whole-genome sequences has exposed the general incidence of the so-called cryptic biosynthetic gene clusters (BGCs), thereby renewing their interest for natural product discovery. As a consequence, genome mining is the often first approach implemented to assess the potential of a microorganism for producing novel bioactive metabolites. By revealing a new level of complexity of natural product biosynthesis, we further illustrate the difficulty of estimation of the panel of molecules associated with a BGC based on genomic information alone. Indeed, we found that the same gene cluster is responsible for the production of compounds which differ in terms of structure and bioactivity. The production of these different compounds responds to different environmental triggers, which suggests that multiplication of culture conditions is essential for revealing the entire panel of molecules made by a single BGC.

## INTRODUCTION

Specialized metabolites are natural products (NPs) that play essential roles by helping the producing strain to cope with various stresses, are used as weapons to outcompete neighboring commensals, or are required at particular physiological or developmental stages (1, 2). Besides their role in improving the fitness of microorganisms in their habitat, among others, molecules emanating from the so-called secondary metabolism are of foremost therapeutic and agroindustrial importance (3, 4). These bioactive compounds are produced by a form of machinery encoded by a group of genes—a biosynthetic gene cluster (BGC)—that, in addition to biosynthetic genes, typically includes genes for expression control, self-resistance, and export (4–6).

Genome sequencing of secondary-metabolite-producing microorganisms has revealed an enormous potential to increase the known chemical space (5), with the promise of new leads in human therapies or for sustainable agriculture. One of the drivers of the renewed interest in NPs was the discovery of so-called cryptic BGCs that are silent under routine laboratory conditions and may therefore specify molecules that had so far been missed during pharmaceutical screening (7–9). An approach that is rapidly gaining momentum is that of expressing cryptic BGCs in a heterologous chassis strain or superhost and exchanging promoter elements within the BGC with those that are expected to result in high levels of expression under laboratory conditions. However, there are several problems associated with this approach. First, it is still difficult to effectively apply it through high-throughput strategies, and it is hard to establish the promise of the activity of a certain BGC on the basis of the DNA sequence alone (10). Second, examples of NPs that are produced from multiple BGCs, or by strains in coculture, are accumulating (11, 12). Alternatively, a single BGC may also be responsible for the production of many (up to over 100) structurally related molecules that differ in terms of their activity (13–15), or BGCs can be associated into “superclusters” that function to produce two or more similar molecules (16, 17). Thus, to optimally exploit the chemical space of NPs, we need to understand the connection between the genomic diversity and the chemical diversity of their biosynthetic pathways.

**FIG 1:**
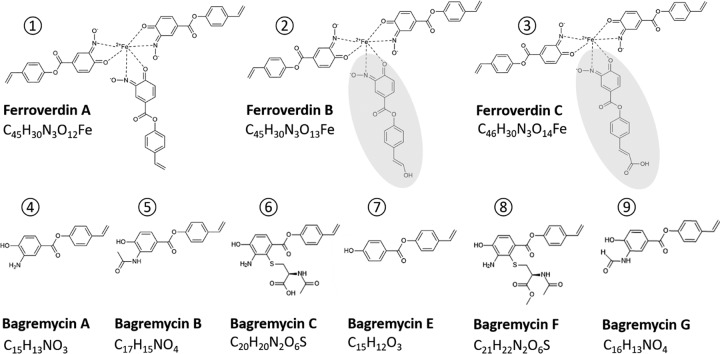
Structures of ferroverdins and bagremycins produced by S. lunaelactis strains. The top line displays the structures of ferroverdins (structures 1 to 3) and the bottom line the structures of bagremycins (structures 4 to 9), including the molecular formula of each. The monomer specific to ferroverdin B (hydroxy-*p*-vinylphenyl-3,4-NHBA) and the monomer specific to ferroverdin C (and carboxy-*p*-vinylphenyl-3,4-NHBA) are shaded in gray.

Here we provide an example that further illustrates the complexity of analyses designed to estimate the metabolome profile as well as the industrial potential of a microorganism based on the genomic information alone. Our work revealed that the ferrous compounds called ferroverdins (Fig. 1, structures 1 to 3 [18–20]), which are inhibitors of cholesterylester transfer protein (CETP) and thus are molecules potentially able to reduce the risk of atherosclerosis (21), and the antibiotics called bagremycins (Fig. 1, structures 4 to 9 [22–24]) are synthesized from the same BGC. These molecules not only have completely different biological functions, but their biosynthesis also requires different environmental triggers. These findings challenge the concept that a given BGC is associated with production of similar compounds in terms of building blocks, structure, bioactivity, and conditions for production.

## RESULTS

### Streptomyces lunaelactis produces ferroverdins and bagremycins.

Strains of Streptomyces lunaelactis, including type strain MM109^T^, were previously isolated from cave moonmilk deposits (25–27). Strain MM109^T^ produces a green pigment whose presence was attributed to the biosynthesis of ferroverdin A (25), a homotrimer of *p*-vinylphenyl-3-nitroso-4-hydroxybenzoate (*p*-vinylphenyl-3,4-NHBA) complexed with one ferrous ion (18, 19). To assess the metabolomic response of S. lunaelactis, including the conditions under which ferroverdin A is produced, strain MM109^T^ was grown under different culture conditions. For this assessment, disc diffusion assays were performed in the presence of a range of different metals.

**FIG 2:**
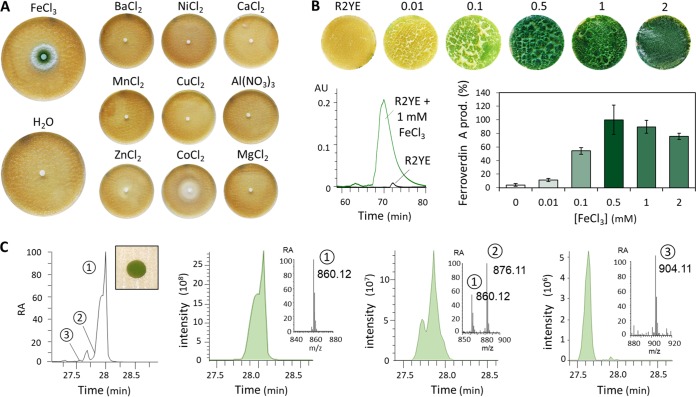
Production of ferroverdins by S. lunaelactis MM109^T^. (A) Paper disc diffusion assays with various metal salts (1 mM). Note that FeCl_3_ was the only metal salt able to trigger the green pigmentation of the mycelium of S. lunaelactis MM109^T^ grown on R2YE agar plates. (B) Induction of ferroverdin production by iron. (Top panels) Phenotypes. (Bottom left panel) HPLC profiles of the crude metabolite extracts. The HPLC profile analyses were performed with crude acetonitrile extracts of S. lunaelactis MM109^T^ grown on R2YE agar plates (black line) or on R2YE medium supplied with 1 mM FeCl_3_ (green line). (Bottom right panel) Semiquantitative analysis of ferroverdin production (Ferroverdin A prod.) by S. lunaelactis MM109^T^ grown on R2YE agar plates supplied with various concentrations of FeCl_3_. The areas of the integrated HPLC peaks of ferroverdin A (observed at 440 nm) were normalized, and the value measured in R2YE plus 0.5 mM FeCl_3_ was fixed at 100%. Bars represent means of results from biological triplicates. AU, arbitrary units. (C) Extracted ion chromatograms (EIC) of the three ferroverdins detected in the full extract of S. lunaelactis MM109^T^. RA, relative abundance.

The results revealed that only the addition of the ferric iron salt FeCl_3_ (as well as the addition of ferrous iron FeSO_4_; data not shown) triggered production of the green pigmented ferroverdin A by S. lunaelactis MM109^T^ (Fig. 2A). Production of ferroverdin A (structure 1 in Fig. 1; see also Fig. 2C) was triggered at a FeCl_3_ concentration as low as 0.01 mM (Fig. 2B). Ultraperformance liquid chromatography-tandem mass spectrometry (UPLC-MS/MS) analysis of extracts of strain MM109^T^ identified an ion peak of *m/z* 876.11 [M^-^], corresponding to ferroverdin B (structure 2 in Fig. 1), and a distinct ion peak of *m/z* 904.11 [M^-^] corresponding to ferroverdin C (structure 3 in Fig. 1). Ferroverdin B and ferroverdin C heterotrimers differ from ferroverdin A in only one of the three ligands used to complex ferrous iron, namely, hydroxy-*p*-vinylphenyl-3,4-NHBA for ferroverdin B and carboxy-*p*-vinylphenyl-3,4-NHBA for ferroverdin C (Fig. 1).

**FIG 3:**
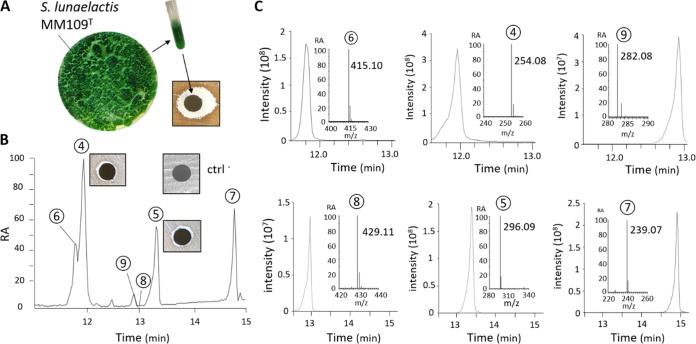
Production of bagremycins by S. lunaelactis MM109^T^. (A) Phenotype of S. lunaelactis MM109^T^ grown on agar plates with R2YE plus 1 mM FeCl_3_ and paper disc diffusion assay assessing the antibacterial activity of the full acetonitrile extract (including both intracellular and extracellular extracts). (B) HPLC-separated fractions of the crude metabolite extract of S. lunaelactis MM109^T^ (RA, relative abundance). Note the paper disc diffusion assay details revealing the antibacterial activity associated with pure bagremycin A (peak 4), and bagremycin B (peak 5); ctrl^-^, paper disc infused with acetonitrile used as negative control (no antibacterial activity). (C) Extracted ion chromatograms (EIC) of the six known bagremycins detected in the full extract of S. lunaelactis MM109^T^.

Under culture conditions allowing the production of ferroverdins, the crude extract of S. lunaelactis MM109^T^ displayed antibacterial activity against Gram-positive bacteria, as exemplified by the observed growth inhibition against the test strain Staphylococcus aureus (Fig. 3A). As pure ferroverdins do not possess antibacterial activities against S. aureus (Fig. 2C, left panel), we undertook the extraction, purification, and identification of the antibacterial metabolites produced by S. lunaelactis. High-pressure liquid chromatography (HPLC) fractionation and subsequent analysis of the active fractions by UPLC-MS/MS identified molecular ion species corresponding to bagremycin A (*m/z* 254.0824 [M-H]^-^), bagremycin B (*m/z* 296.0931 [M-H]^-^), bagremycin C (*m/z* 415.0974 [M-H]^-^), bagremycin E (*m/z* 239.0714 [M-H]^-^), bagremycin F (*m/z* 429.1133 [M-H]^-^), and bagremycin G (*m/z* 282.0777 [M-H]^-^) (Fig. 3B and C; see also structures 4 to 9 in Fig. 1). Bagremycins are amino-aromatic antibiotics resulting from the condensation of 3-amino-4-hydroxybenzoic acid (3,4-AHBA) with *p*-vinylphenol (22–24).

**FIG 4:**
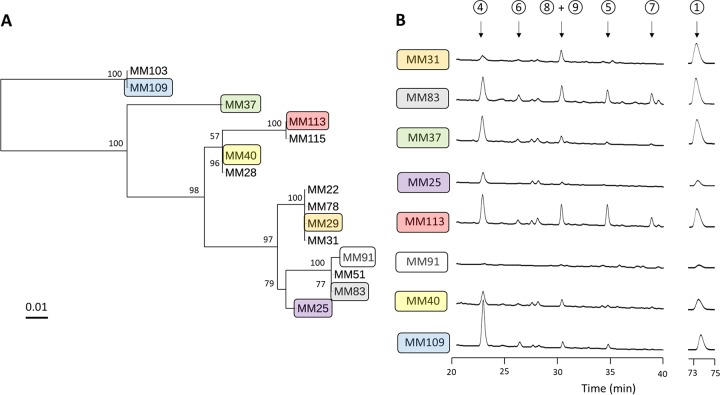
Production of ferroverdins and bagremycins by different S. lunaelactis strains. (A) Multilocus sequence analysis (MLSA) of moonmilk isolates belonging to the species S. lunaelactis. (B) HPLC profiles at the indicated retention times were determined for monitoring bagremycin and ferroverdin production by eight different S. lunaelactis strains grown on ISP7 medium.

MM109^T^ is the type strain of S. lunaelactis, but many other strains belonging to the species S. lunaelactis were isolated from different moonmilk deposits. Multilocus sequence analysis (MLSA) performed on 15 independently isolated S. lunaelactis strains grouped them into different clusters (Fig. 4A), and one representative of each cluster was investigated for the production of bagremycins and ferroverdins. The crude extracts of the selected eight different representative strains (MM25, MM31, MM37, MM40, MM83, MM91, MM109, and MM113) were analyzed by HPLC, which revealed that all strains except strain MM91 were able to produce at least one bagremycin and one ferroverdin (Fig. 4B). These data suggest that the production of bagremycins, like the production of ferroverdins, is a common feature of moonmilk-dwelling strains belonging to the species S. lunaelactis.

### Identification of a BGC similar to *fev* and *bag* clusters in S. lunaelactis MM109^T^.

**FIG 5:**
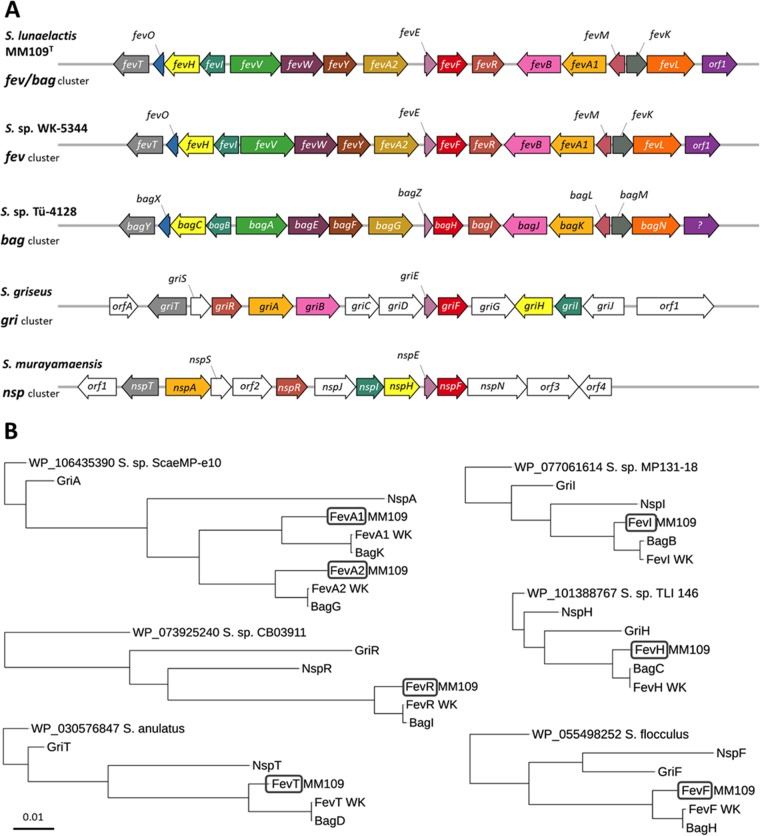
Comparative analysis of known BGCs involved in the production of nitroso-aromatic and amino-aromatic metabolites in *Streptomyces* spp. (A) Genetic organization of the BGCs involved in ferroverdin (*fev*), bagremycin (*bag*), 4-hydroxy-3-nitrosobenzamide (*nsp*), and grixazone (*gri*) production in *Streptomyces* spp. See Table 1 for the known function and/or predicted function associated with the product of each gene. (B) Phylogeny analysis of proteins conserved among the ferroverdin (*fev*), bagremycin (*bag*), 4-hydroxy-3-nitrosobenzamide (*nsp*), and grixazone (*gri*) BGCs. The trees were rooted by including as outgroup the proteins most similar to S. lunaelactis MM109^T^ proteins.

In order to identify the genes involved in the production of bagremycins (*bag*) and/or ferroverdins (*fev*) in S. lunaelactis MM109^T^, the genome of this strain (28) was mined using antiSMASH v4.0 software (29). A total of 37 BGCs were identified, specifically, 36 BGCs on the linear chromosome and 1 additional BGC on linear plasmid pSLUN1 (see Fig. S1 in the supplemental material). BGC 12 (from S. lunaelactis 21350 [SLUN_21350] to SLUN_21430 [28]) exhibits strong gene synteny with the bagremycin BGC from *Streptomyces* sp. Tü 4128 (30, 31), with high similarity between the predicted gene products (coverage, 99%; average identity, 86.2%; average similarity, 91.9%) (Fig. 5A) (Table 1). Surprisingly, BGC 12 displayed similarly strong gene synteny and high similarity between its gene products and those seen with the *fev* cluster (GenBank accession no. AB689797) of the ferroverdin producer *Streptomyces* sp. WK-5344 (coverage, 99%; average identity, 85.8%; average similarity, 91.3%) (Fig. 5A) (Table 1). The levels of identity between the proposed *fev* cluster of *Streptomyces* sp. WK-5344 and the *bag* cluster of *Streptomyces* sp. Tü 4128 were even greater, with (on average) 96% amino acid (aa) identity and 97.4% similarity (Fig. 5A) (Table 1). The exceptionally high levels of average amino acid identity suggest that biosynthesis of bagremycins and biosynthesis of ferroverdins might be mediated by the same BGC. This conjecture was further supported by the results of comparative analysis of the BGCs involved in the production of other amino/nitroso-aromatic metabolites, i.e., the *nsp* gene cluster for biosynthesis of 4-hydroxy-3-nitrosobenzamide in Streptomyces murayamaensis (32) and the *gri* gene cluster involved in grixazone production in Streptomyces griseus (33). The *nsp* and *gri* clusters share seven homologous genes with those identified in the *bag*/*fev* cluster (Fig. 5A) (Table 1), specifically, those encoding (i) the *Streptomyces* antibiotic regulatory protein (SARP)-family transcriptional activators (BagI, FevR, NspR, and GriR), (ii) the LuxR-family transcription regulators (BagY, FevT, NspT, and GriT), (iii) the 3,4-AHBA synthases (BagC, FevH, NspH, and GriH), (iv) the DhnA-type aldolase (BagB, FevI, NspI, and GriI), (v) the o-aminophenol oxidases (BagH, FevF, NspF, and GriF), (vi) the copper chaperones (BagZ, FevE, NspE, and GriE), and (vii) the flavin adenine dinucleotide (FAD)-dependent oxygenases (BagK/G. FavA1/A2, NspA, and GriA). Phylogeny analyses revealed that Fev and Bag proteins always branched together in clusters separated from Nsp and Gri proteins (Fig. 5B).

**TABLE 1:** Elements of the ferroverdin/bagremycin BGC in *S. lunaelactis* and comparative analysis of similar clusters in other *Streptomyces* species^*a*^

Gene ID (SLUN)	Name^*b*^	Predicted function	*fev* ID in *Streptomyces* sp. WK-5344	*bag* ID in *Streptomyces* sp. Tü-4128; accession no.	*gri* in *S. griseus* subsp. *griseus*	*nsp* in S. murayamaensis
21345		Aminotransferase	NA	23415 (NS); WP_122618577	NF	NF

21350	ORF1	Prephenate dehydrogenase	89–93; BAM73624	*Pseudo*-23410	NF	NF

21355	*fevL*	Decarboxylase	88–93; BAM73625	88–93 (*bagN*); 23405/ WP_122618385	NF	NF

21360	*fevK*	Decarboxylase	82–89; BAM73626	82–89 (*bagM*); 23400/ WP_122618384	NF	NF

21365	*fevM*	Transcriptional regulator, MarR family	90–92; BAM73627	90–92 (*bagL*); 23395/ WP_122618383	NF	NF

21370	*fevA1*	FAD-dependent oxygenase	79–86; BAM73628	78–86 (*bagK*); 23390/ WP_122618382	57–69 (*griA*); BAF36643	51–65 (*nspA*); BAJ08166

21375	*fevB*	Putative bagremycin transporter	88–93; BAM73629	88–93 (*bagJ*); 23385/ WP_122618381	57–71 (*griB*); BAF36644	NS (*orf2*); BAJ08168

21380	*fevR*	SARP-family transcriptional activator	81–87; BAM73630	80–86 (*bagI*); 23380/ AKA27633.1	47–64 (*griR*); BAF36642	53–68 (*nspR*); BAJ08169

21385	*fevF*	*o*-Aminophenol oxidase	89–93; BAM73631	89–93 (*bagH*); 23375/ WP_122618379	68–78 (*griF*); BAF36648	60–74 (*nspF*); BAJ08174

21390	*fevE*	Copper chaperon	73–79; BAM73632	*Pseudo*-23370 (*bagZ*)	61–74 (*griE*); BAF36647	57–68 (*nspE*); BAJ08173

21395	*fevA2*	FAD-dependent oxygenase	83–90; BAM73633	83–90 (*bagG*); 23365/ WP_122618378	56–69 (*griA*); BAF36643	52–65 (*nspA*); BAJ08166

21400	*fevY*	Phospho-2-dehydro-3- deoxyheptonate aldolase	88–94; BAM73634	88–94 (*bagF*); 23360/ WP_122618377	NF	NF

21405	*fevW*	Bagremycin synthetase	84–89; BAM73635	84–89 (*bagE*); 23355/ WP_122618576	NF	NF

21410	*fevV*	Tyrosine ammonia-lyase	92–95; BAM73636	91–95 (*bagA*); 23350/ WP_122618575	NF	NF

21415	*fevI*	DhnA-type aldolase	91–96; BAM73637	91–96 (*bagB*); 23345/ WP_122618376	72–85 (*griI*); BAF36651	74–85 (*nspI*); BAJ08171

21420	*fevH*	3,4-AHBA synthase	92–96; BAM73638	92–96 (*bagC*); 23340/ WP_122618375	77–87 (*griH*); BAF36650	79–89 (*nspH*); BAJ08172

21425	*fevO*	Hypothetical protein	86–95; BAM73639	85–94; 23335/ WP_122618374	NF	NF

21430	*fevT*	LuxR-family transcriptional regulator	84–92; BAM73640	84–92 (*bagY*); 23330/ WP_122618373	63–78 (*griT*); BAF36640	62–76 (*nspT*); BAJ08165

21435	ORF2	Carboxymuconolactone decarboxylase	NS; BAM73641	*Pseudo*-23325	NF	NF

a *bag*, bagremycin cluster (NZ_QTSY01000069) (DXM28_RS); *fev*, ferroverdin cluster (AB689797); *gri*, grixazone cluster (AB259663); *nsp*, nitrosobenzamide cluster (AB530136). Abbreviations: ID, identifier; sim, similarity; NA, sequence not available; NS, not similar; NF, none found.

b Gene names were provided based on the *fev* cluster of *Streptomyces* WK-5344 (GenBank accession no. AB689797.1). Paired numbers refer to the percentage of amino acid identity and similarity, respectively.

10.1128/mBio.01230-19.1 FIG S1 Schematic representation of S. lunaelactis MM109^T^ chromosome and its two extrachromosomal plasmids. (A) Chromosome. From the outside to the inside, the concentric tracks represent the following: nucleotide position; predicted BGCs with associated labels; coding DNA sequences (CDSs) on the forward strand; CDSs on the reverse strand; tRNA (orange) and rRNA (magenta) genes; per-base read coverage (green and red for above and below average, respectively; window size = 10,000; base step size = 1,000); GC content (GC%; window size = 10,000; base step size = 1,000); GC skew ([G + C]/[G − C], window size = 8,396; base step size = 839); cumulative GC skew. The position of the predicted origin of replication (OriC) is indicated. (B) Linear plasmid pSLUN1. (C) Circular plasmid pSLUN2. The same track coloring applies for the plasmids. (The coverage and GC content sliding averages have a window size of 1,000 bp and a base step size of 100 bp). Download FIG S1, TIF file, 1.6 MB. Copyright © 2019 Martinet et al. 2019 Martinet et al. This content is distributed under the terms of the Creative Commons Attribution 4.0 International license.

Finally, only one single BGC is similar to the *fev* and *bag* gene clusters in S. lunaelactis MM109^T^ as well as in the genomes of 16 other S. lunaelactis strains. This makes it extremely unlikely that bagremycins and ferroverdins should be specified by two different BGCs. A plausible pathway for the synthesis of both bagremycins and ferroverdin A from the *fev*/*bag* cluster is proposed in the Discussion (see also Fig. 8).

### Inactivation and/or duplication of *fevR* affects both ferroverdin and bagremycin production.

**FIG 6:**
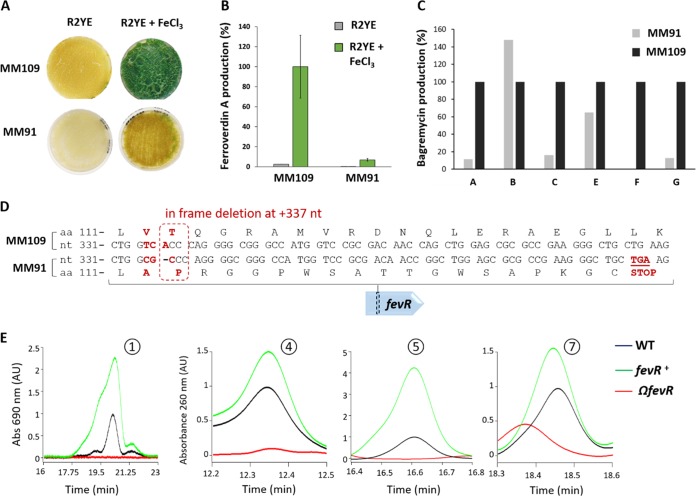
FevR (BagI) is involved in the production of both ferroverdin A and bagremycins. (A) Phenotypes of S. lunaelactis strains MM109^T^ and MM91 grown in media activating (R2YE plus FeCl_3_) or not inducing (R2YE) the production of ferroverdin A. (B) Semiquantitative analysis (HPLC) of ferroverdin A produced by these strains. (C) Semiquantitative analysis (HPLC) of bagremycins produced by S. lunaelactis strains MM109^T^ and MM91. (D) Identification in the *fev* cluster of S. lunaelactis strain MM91 (accession number MG708299) of the in-frame deletion at nt position +337 of *fevR* encoding the SARP-family transcription activator. (E) Details of HPLC profiles focused on peaks associated with bagremycin A, bagremycin B, bagremycin F, and ferroverdins from extracts of mutant strains of S. lunaelactis MM109^T^ in which *fevR* is inactivated (Ω*fevR*) or with one supplementary copy of *fevR* (*fevR*^+^). Abs, absorbance. WT, wild type.

Among the S. lunaelactis strains that were collected from the moonmilk samples, strain S. lunaelactis MM91, which does possess a complete *fev*/*bag* cluster (GenBank accession no. MG708299), failed to produce the ferroverdin-associated green pigmentation under conditions of growth in iron-containing media (Fig. 6A and B).

MM91 also showed reduced production of all bagremycins except bagremycin B (Fig. 6C). The phenotype of S. lunaelactis MM91 allowed a forward genetic approach to assess if the *fev* cluster is indeed responsible for production of both bagremycins and ferroverdins. Genome sequencing and single nucleotide polymorphism (SNP) analysis of S. lunaelactis strain MM91 revealed a deletion at nucleotide (nt) position +337 of SLUN_21380 (*bagI* or *fevR*), which encodes the likely pathway-specific activator of the BGC (Fig. 6D). This deletion resulted in a frameshift leading to a premature stop codon, 17 amino acids downstream of the deletion, and thus in a truncated (and likely inactive) protein.

To ascertain whether FevR/BagI is required for both ferroverdin and bagremycin production, the SLUN_21380 gene was first interrupted in strain S. lunaelactis MM109^T^ by insertion of a thiostreptone resistance cassette. Introduction of plasmid pBDF028 into spores of MM109^T^ allowed isolation of thiostreptone-resistant clones in which occurred a single recombination event corresponding to a 791-bp internal fragment of SLUN_21380 (*fevR*/*bagI*). The single-crossover event resulted in a duplication of *fevR*, with the first copy lacking the last 86 nt, resulting in a truncated FevR protein, and the second copy lacking the promoter region and the first 116 nt. One clone (referred to as strain Ω*fevR* here, where “Ω” represents inactivation by interruption) was selected for further analyses. Strain Ω*fevR* displayed loss of the ferroverdin-associated green pigmentation under conditions of growth on iron-supplemented R2YE medium. HPLC analysis of the crude extracts of strain Ω*fevR* confirmed the absence of ferroverdins and bagremycins (Fig. 6E). These data suggest that FevR/BagI is indeed the pathway-specific activator for the BGC, and this interpretation was further supported by the observation that introduction of an additional copy of *fevR*/*bagI* into S. lunaelactis MM109^T^ resulted in increased production of both ferroverdins and bagremycins (Fig. 6E). Finally, complementation of Ω*fevR* (strain MM109BD3; Table 2) by the low-copy-number plasmid harboring an intact copy of *fevR* with its own promoter allowed restoration of production of both ferroverdin and bagremycin (not shown).

**TABLE 2:** Plasmids and strains used in this study^*a*^

Plasmids and strains	Description	Source or reference(s)
Plasmids		
pJET1.2/blunt	E. coli plasmid used for high-efficiency blunt-end cloning of PCR products (Amp^r^)	Thermo Scientific
pBDF019	pJET1.2 derivative containing the *slun21380* gene (*fevR*) and its upstream (217-bp) and downstream (360-bp) regions (Amp^r^)	This study
pSET152	Integrative vector transmissible by conjugation from E. coli to *Streptomyces* spp. [*lacZα*, *ori* (pUC18), *aac(3)IV* (Apra^r^), *oriT* (RK2), *attP* (ØC31), *int* (ØC31)]	42
pBDF021	pSET152 derivative containing the insertion of pBDF019 cloned in EcoRI and XbaI sites (Apra^r^)	This study
pBDF027	pJET1.2 derivative containing an internal fragment (791 bp amplified with *fevR*+118f_*Xba*I and *fevR*+909r_*Pst*I primers) of the *slun21380* (*fevR*) coding sequence (Amp^r^)	This study
pSET151	Nonreplicating plasmid in *Streptomyces* spp. [*lacZα*, *oriT* (RK2), *xylE*, *tsr* (Thio^r^), *bla* (Amp^r^), *ori* (pUC19)]	42, 43
pBDF028	pSET151 derivative containing the insertion of pBDF027 cloned in XbaI and PstI sites (Amp^r^, Thio^r^)	This study
pUZ8002	Nontransmissible plasmid supplying transfer functions for mobilization of *ori*T-containing vectors from E. coli to *Streptomyces* spp. (Kan^r^)	41
pAU3-45	pSET152 derivative with a thiostrepton resistance gene inserted into the blunted NheI restriction site [*lacZα*, *ori* (pUC18), *aac(3)IV* (Apra^r^), *oriT* (RK2), *attP* (ØC31), *int* (ØC31), *tsr* (Thio^r^)]	44
pBDF029	pBDF022 derivative containing the apramycin resistance cassette from pIJ773 cloned in PstI and HindIII sites (Thio^r^, Amp^r^, Apra^r^)	This study
pIJ773	pBluescript II SK(+)-based plasmid containing the apramycin resistance cassette [*oriT* (RK2) + *aac(3)IV* (Apra^r^)] flanked by FRT (FLP recognition target) recombination sites	43
pBDF022	pHJL401 derivative containing the insert of pBDF019 cloned in EcoRI and XbaI sites (Thio^r^, Amp^r^)	This study
pHJL401	Intermediate-copy-number (∼10 copies) vector in *Streptomyces* spp. [*lacZα*, *ori* (pUC19), *ori* (SCP2), *tsr* (Thio^r^), *bla* (Amp^r^)]	42

Strains		
E. coli DH5α	General cloning host	Gibco-BRL
E. coli ET12567	Nonmethylating (*dam*, *dcm*, *hsdS* null mutants) host for transfer of plasmid DNA into *Streptomyces* spp., used together with pUZ8002 (Cml^r^, Tet^r^)	45
Streptomyces lunaelactis MM109^T^	Wild-type and reference strain of Streptomyces lunaelactis	25
Streptomyces lunaelactis MM91	Wild-type strain Streptomyces lunaelactis MM91	26
Streptomyces lunaelactis MM109BD1 (Ω*fevR*)	MM109 derivative with pBDF028 inserted to disrupt gene *slun21380* (*fevR*) (Thio^r^)	This study
Streptomyces lunaelactis MM109BD2 (*fevR*^+^)	MM109^T^ derivative with pBDF021 inserted to add an additional copy of *slun21380* (*fevR*) controlled by its native promoter (Apra^r^)	This study
Streptomyces lunaelactis MM109BD3	Complementation of strain MM109BD1 (Ω*fevR*) with pBDF029 (Thio^r^, Apra^r^)	This study

a Amp^r^, ampicillin resistance; *lacZα*, LacZ galactosidase alpha subunit coding sequence; *ori* (pUC18/pUC19), origin of replication of the pUC18/pUC19 plasmid (E. coli); *aac(* 3*)IV*, apramycin resistance marker; Apra^r^, apramycin resistance; *oriT* (RK2), origin of conjugative transfer from plasmid RK2; *attP* (ØC31), phage attachment site of the ØC31 integrase [= *int* (ØC31)]; *xylE*, catechol 2,3-dioxygenase (reporter gene); *tsr*, thiostrepton resistance gene; Thio^r^, thiostrepton resistance; *bla*, ampicillin resistance gene; *dam*, *dcm*, *hsdS*, genotype of methylase-deficient E. coli strain; Cml^r^, chloramphenicol resistance; Tet^r^, tetracycline resistance.

### Iron is required for production of *p*-vinylphenyl-3,4-NHBA but its presence not mandatory for bagremycin biosynthesis.

**FIG 7:**
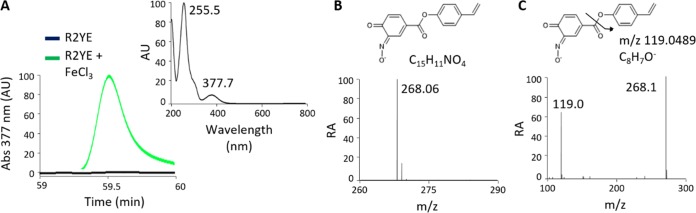
Iron supply is mandatory for production of *p*-vinylphenyl-3-nitroso-4-hydroxybenzoate. (A) (Left panel) HPLC chromatogram showing the identification of *p*-vinylphenyl-3-nitroso-4-hydroxybenzoate in the extracts of S. lunaelactis MM109 grown on R2YE plus 1 mM FeCl_3_ (green curve) and the (right panel) absorbance spectrum from 200 to 800 nm of *p*-vinylphenyl-3-nitroso-4-hydroxybenzoate. Note the absence of the compound when S. lunaelactis MM109 is grown on R2YE (black line). (B) Mass spectrum of the compound present in the chromatographic peak and its proposed structure. (C) MS/MS spectra of *p*-vinylphenyl-3-nitroso-4-hydroxybenzoate with the proposed fragmentation mechanism.

If ferroverdins are detected only when iron is supplied in the cultivation media, a key issue that remains unaddressed is whether iron is required only for complexation of the *p*-vinylphenyl-3,4-NHBA monomers (and/or the other monomers required for ferroverdin B and C) or whether iron is necessary for the synthesis of *p*-vinylphenyl-3,4-NHBA, the monomer of ferroverdins. An HPLC chromatogram of extracts of S. lunaelactis MM109^T^ grown on R2YE and R2YE supplied with 1 mM FeCl_3_ revealed the presence of the *p*-vinylphenyl-3,4-NHBA monomer only when strain MM109^T^ was grown under conditions of high levels of iron (Fig. 7A). HPLC fractionation and subsequent UPLC-MS/MS analysis confirmed the presence of a molecular ion species that corresponded to *p*-vinylphenyl-3,4-NHBA (*m/z* 268.06 [M^-^]) (Fig. 7B and C). Instead, bagremycins were detected under both sets of conditions (Fig. S2), suggesting that, in contrast to the results seen with *p*-vinylphenyl-3,4-NHBA, iron overload is not mandatory for their biosynthesis. Thus, iron is not only required for complexation of the three *p*-vinylphenyl-3,4-NHBA ligands with ferrous ion and for generation of ferroverdin A but is also necessary for their biosynthesis.

10.1128/mBio.01230-19.2 FIG S2 Identification of bagremycin A (left graph), bagremycin B (middle graph), and bagremycin G (right graph) in the culture extracts of Streptomyces lunaelactis MM109 grown on R2YE (black curve) and on R2YE plus 1 mM FeCl_3_ (green curve). Download FIG S2, TIF file, 0.2 MB. Copyright © 2019 Martinet et al. 2019 Martinet et al. This content is distributed under the terms of the Creative Commons Attribution 4.0 International license.

## DISCUSSION

In this work, we provide genomic, genetic, and metabolomic evidence that a single BGC (*fev*/*bag*) is responsible for the synthesis of both ferroverdins and bagremycins. That a single BGC is responsible for the production of several related compounds is not unusual, but several features make the case of the *fev*/*bag* BGC unique. For example, the chemical compositions of ferroverdins and bagremycins (nitroso-aromatic versus amino-aromatic compounds) are different, as are their structural organizations (trimers versus monomers), whereas these two characteristics are conserved in other BGCs producing multiple compounds. But what makes this biosynthetic pathway exceptional is that the bagremycins and *p*-vinylphenyl-3,4-NHBA, the monomer of ferroverdins, are not produced under the same culture conditions. Indeed, excess iron supply is mandatory for *p*-vinylphenyl-3,4-NHBA production and for its subsequent complexation to a tripartite molecule, while the bagremycins are produced under conditions of low iron levels. Thus, iron is a key molecule that determines the distinct biological activities produced by S. lunaelactis: bagremycins display antibacterial, antifungal, and antiproliferative activities, presumably in order to inhibit growth of neighboring competing bacteria and fungi and/or to control self-proliferation, while *p*-vinylphenyl-3,4-NHBA production would be required (to limit iron-mediated oxidative damage) only when iron excess constitutes a main threat. The change of the amino in a nitroso group in bagremycin allows complexation with iron, which strongly suggests an evolutionary driver for this chemical diversification.

**FIG 8:**
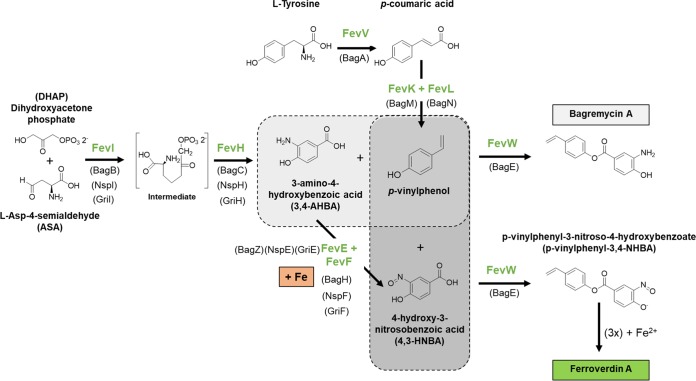
Proposed pathway for biosynthesis of bagremycin and ferroverdin.

We propose a possible biosynthetic pathway for both bagremycins and ferroverdins (Fig. 8) on the basis of the deduced function of the different components of the *bag/fev* clusters and of the current knowledge on the biosynthetic pathway of bagremycins (22, 30, 31, 34). In addition, as biosynthesis is known to occur for two other types of amino/nitroso-aromatic compounds, namely, grixazones (*gri* genes) and 4-hydroxy-3-nitrosobenzamide (*nsp* genes), the available information regarding these BGCs is useful to predict steps shared by the studied pathways.

As demonstrated previously, bagremycin A is derived from precursors *p*-vinylphenol and 3-amino-4-hydroxybenzoic acid (3,4-AHBA) (22). The *p*-vinylphenol precursor is generated in two steps from l-tyrosine, with the first step consisting of deamination by FevV/BagA in *trans*-coumaric acid and the second step being decarboxylation in *p*-vinylphenol by FevK (BagM) and FevL (BagN). Indeed, FevK and FevL form a UbiX-UbiD system in which UbiD is a decarboxylase that requires prenylated flavin mononucleotide (FMN) as a cofactor, which is provided by the flavin prenyltransferase UbiX (35). Regarding the second precursor, 3,4-AHBA, it was demonstrated previously that it is formed from dihydroxyacetone phosphate (DHAP) and l-aspartic-4-semialdehyde (ASA) through two reactions catalyzed by BagB (FevI) and BagC (FevH) (30). Condensation of *p*-vinylphenol and 3,4-AHBA to form bagremycin A would be mediated by FevW, which shows high levels of similarity to proteins of the phenylacetate-CoA ligase family, which catalyze the condensation of two molecules by their carboxylic acid and alcohol moieties through adenylation.

Regarding the synthesis of *p*-vinylphenyl-3,4-NHBA, the monomer of ferroverdin A and a major monomer of other ferroverdins, we propose that it would directly result from precursors *p*-vinylphenol and 4-hydroxy-3-nitrosobenzoic acid (4,3-HNBA) (Fig. 7). Direct generation of 4.3-HNBA would occur via the nitrosation of 3,4-AHBA and would be mediated by the copper-containing oxidase FevF (with FevE as chaperone). FevF is a copper-containing oxidase which is orthologous to NspF and GriF, while FevE is a copper chaperone orthologous to NspE and GriE (Table 1). NspE bring two coppers to NspF, which converts 3-amino-4-hydroxybenzamide to 4-hydroxy-3-nitrosobenzamide in S. murayamaensis (32, 36). Likewise, GriE donates two coppers to GriF, which converts *o*-aminophenol into *o*-quinone imine in Streptomyces griseus (37). Since these FevF/FevE orthologous proteins mediate the oxidation of 3.4-AHBA-like substrates to generate quinone imine moieties which would be consecutively nitrosylated, we suggest that both of these enzymes are able to mediate the transformation of 3,4-AHBA to 4,3-HNBA. Condensation of 4,3-HNBA with *p*-vinylphenol by FevW would result in the formation of *p*-vinylphenyl-3-nitroso-4-hydroxybenzoate, the monomer of ferroverdins.

Finally, in addition to the role of iron as a vital element for growth (38, 39), morphogenesis, and metabolite production (12, 39, 40), our work reveals an unprecedented complexity of natural product biosynthetic pathways where this element is also an environmental trigger able to change the pattern of natural compounds produced by a single BGC. By revealing an exception to the concept that a single BGC should produce a single type of bioactive molecules, our results also highlight the difficulty of estimating the metabolic potential of an organism on the basis of genomic information alone.

## MATERIALS AND METHODS

### Strains and culture conditions.

All strains used in this study are listed in Table 1. Escherichia coli strains were grown in liquid LB or on solid LB medium agar plates and incubated at 37°C. Media used for solid *Streptomyces* cultures were R2YE, SFM, and ISP7 agar plates, which were incubated at 28°C. When required, the antibiotics thiostreptone, kanamycin, nalidixic acid, and ampicillin were added in the culture media. The type strain S. lunaelactis MM109^T^ (25) and other S. lunaelactis strains were isolated from the cave “Grotte des Collemboles’ Collemboles” (Comblain-au-Pont, Belgium) (25–27). The medium compositions used and the methods associated with *Streptomyces* species were all as described previously (41).

### Creation of *fevR* knockout and overexpression mutants in S. lunaelactis MM109.

In order to assess the effect of *fevR*/*bagI* on production of both bagremycin and ferroverdin, we first generated a S. lunaelactis MM109 knockout strain in which *orf* SLUN21380 was interrupted by a single-crossover event with insertion of a thiostreptone resistance cassette. A 791-bp internal fragment (starting at position nt +116 and lacking the last 81 nt of SLUN21380) was amplified by PCR using primers *fevR*+118f_*Xba*I (TCTCTAGACGAACCAGGTGGTCAGCC) and *fevR*+909r_*Pst*I (CGCTGCAGGTCGTTCTCCAGGCGC) (the underlined characters represent restriction sites). The PCR product was cloned into plasmid pJET1.2 (Blunt cloning vector; Thermo Fisher Scientific), and the product was named pBDF027 and sequenced for verification of the amplified PCR fragment. This plasmid was then digested by XbaI and PstI-HF restriction enzymes (NEB), and the *fevR* insertion was subsequently cloned into pSET151 (41) digested with the same enzymes. The plasmid resulting from the ligation, named pBDF028, was introduced into the S. lunaelactis MM109 chromosome by intergeneric conjugative transfer using methylation-deficient Escherichia coli ET12567 containing the plasmid pUZ8002 (ETpUZ) as conjugation partner. After growth of E. coli ETpUZ to an optical density (OD) of 0.4 (50 ml of culture in a mixture of LB plus chloramphenicol [30 μg/ml] plus kanamycin [50 μg/ml] plus ampicillin [100 μg/ml]), cells were washed to remove antibiotics and mixed with S. lunaelactis spores for mating. Selection of exconjugants was carried out on soy flour mannitol (SFM) medium (plus 10 mM MgCl_2_) overlaid with thiostrepton (50 μg/ml) and nalidixic acid (25 μg/ml) after 16 h of incubation at 28°C. The single-crossover insertion of pBDF028 into the *fevR* locus was confirmed by PCR, and one selected clone was named Ω*fevR* (MM109BD1) for further analyses.

To generate a S. lunaelactis strain overproducing FevR, an entire copy of *fevR* (slun21380) that included the 217-bp upstream and 360-bp downstream regions was amplified by PCR with primers BDF53 (GATCTAGAAAGCTTGGCTCTGTCCAGTGAGACATCC) and BDF54 (GCGAATTCGTACTCGATGTCACCCGCC) to obtain a 1,570-bp fragment flanked by EcoRI and XbaI restriction sites. The PCR product was first cloned into pJET1.2, and the product was named pBDF019. Using EcoRI-HF and XbaI enzymes, the 1,570-bp *fevR*-containing fragment was cloned into a pSET152 (41) integrative vector, producing pBDF021. Conjugation was performed with spores (10^8^) of S. lunaelactis MM109 on SFM (plus 10 mM MgCl_2_) overlaid with apramycin and nalidixic acid. Integration of the plasmid was confirmed by PCR with pSET_forward (GAGCGGATAACAATTTCACACAGGA) and BDF53 or pSET_reverse (CGCCAGGGTTTTCCCAGTCACGAC) and BDF54 primers. The selected MM109^T^ derivative with pBDF021 inserted to add an additional copy of *slun21380* (*fevR*) controlled by its native promoter was named MM109BD2 (Table 2).

For complementation of the Ω*fevR* mutant (strain MM109BD1), an entire copy of *fevR* was excised from pBDF019 using EcoRI and XbaI restriction sites and cloned in the same sites of the intermediate-copy-number pHJL401 plasmid (∼10 copies) was achieved by conjugation with plasmid pBDF029, leading to production of pBDF022. To include in pBDF022 the origin of transfer required for conjugation and a resistance marker for selection of exconjugants, the apramycin resistance cassette (from pIJ773) containing both *oriT* (RK2) and *aac(3)IV* (ApraR) was amplified by PCR with primers Apra_P1 (ATTCCGGGGATCCGTCGACC) and Apra_P2_HindIII (AAAAAGCTTTGTAGGCTGGAGCTGCTTC) and cloned in the same sites of pBDF022, resulting in plasmid pBDF029. Conjugation of pBDF29 into spores (10^8^) of S. lunaelactis MM109BD1 was performed as described above.

### Compound extraction and analysis by high-pressure liquid chromatography (HPLC).

Compound extraction and HPLC analysis were mainly performed as described previously (26). All S. lunaelactis strains were cultured for 10 days on different solid media on petri dishes (90-mm diameter, 25 ml of medium) and incubated at 28°C. The solid cultures were cut into small pieces (about 0.5 by 0.5 cm) and then mixed overnight with an equal volume (25 ml per plate) of ethyl acetate. The mixture was centrifuged (20 min at 4,000 rpm) and the supernatant was evaporated (25°C at 210 rpm) on a rotary evaporator (IKA RV10 digital; VWR, Radnor, PA, USA). The dried crude extract was resuspended in 1 ml of acetonitrile for further analyses. The full extract was then fractionated by HPLC (Waters, Milford, MA, USA) using a Waters 2695 Separations Module (Alliance) with a Waters 2998 photodiode array detector coupled to a Waters Fraction Collector (model WFC III). The extracts were analyzed on a Luna Omega PS C_18_ column (Phenomenex) (2.1 mm by 150 mm, 5-μm particle size, 100-Å pore size) at a column temperature of 40°C. Extract separation was achieved by increasing the ratio of acetonitrile (Barker; HPLC Far UV grade) to water (MilliQ filtration using a pore size of 0.22 μm)–0.05% trifluoroacetic acid (TFA) (Thermo Fisher Scientific, San Jose, CA, USA; sequencing grade) (from 0% to 100% acetonitrile during 80 min) at a flow rate of 450 μl/min. Online UV absorption measurement was performed from 190 to 800 nm. Data were analyzed using Empower 3 software (Waters, Milford, MA, USA) and Xcalibur v2.2 software (Thermo Fisher Scientific, San Jose, CA, USA). Fractions were subsequently tested for antibacterial activities against Staphylococcus aureus (ATCC 25923) by disk diffusion assay as described previously (26).

### Compound identification by ultraperformance liquid chromatography-tandem mass spectrometry (UPLC-MS/MS).

UV light-visible light absorbance spectra were obtained by analytical HPLC RP-C_18_ analyses. High-resolution electrospray ionization mass spectrometry (HRESIMS) data were acquired on a Q Exactive Plus hybrid Quadrupole-Orbitrap mass spectrometer (Thermo Fisher Scientific, San Jose, CA, USA). Briefly, compounds were separated by reverse-phase chromatography using ultraperformance liquid chromatography (UPLC I-Class; Waters) and a Nucleodur C_18ec_ column (Macherey-Nagel) (2.0 mm by 150 mm, 5-μm particle size). Elution was achieved by increasing the ratio of acetonitrile to water (MilliQ filtration using a pore size of 0.22 μm)–0.05% trifluoroacetic acid (for positive-ionization mode) (from 0% to 62.5% during 30 min and then from 62.5% to 100% during 8 min) at a flow rate of 300 μl/min. For the negative-ionization mode, elution was achieved by increasing the ratio of acetonitrile to water (MilliQ filtration using a pore size of 0.22 μm)–0.1% formic acid (from 0% to 62.5% during 30 min and then from 62.5% to 100% during 8 min) at a flow rate of 300 μl/min on a Luna Omega PS C_18_ column (Phenomenex) (2.1 mm by 150 mm, 5-μm particle size, 100-Å pore size). On-line UV absorption measurement was performed at 210 and 265 nm, and the chromatography system was finally coupled to a Q Exactive Plus hybrid Quadrupole-Orbitrap mass spectrometer (Thermo Fisher Scientific, San Jose, CA, USA), operated in positive-ion mode (for bagremycins) and in negative mode (for ferroverdins and bagremycins), and programmed for data-dependent acquisitions. Survey scans were acquired at a mass-resolving power level of 140,000 FWHM (full width at half-maximum) and at 100 to 1,500 *m/z* (accumulation target, 1 × 10^6^ ion). The five ions showing the greatest intensity were then selected for tandem mass spectrometry (MS/MS) experiments by the use of higher-energy collision dissociation (HCD) fragmentations and stepped normalized collision energy (NCE) (21,2; 25; 28) within 2-atomic-mass-unit (amu) isolation windows (resolution, 17,500; accumulation target, 1 × 10^5^ ions). Dynamic exclusion was enabled for 10 s. Data were analyzed using Xcalibur v2.2 (Thermo Fisher Scientific, San Jose, CA, USA).

Each of the compounds was identified in accordance with its exact mass and isotope pattern, observation of the MS/MS spectra obtained by molecular ion fragmentation, and the UV-VIS absorbance spectra. For each molecule, a fragmentation pathway was proposed (see Fig. S3 in the supplemental material).

10.1128/mBio.01230-19.3 FIG S3 ESI (negative)-MS/MS spectra and proposed fragmentation mechanisms for each deprotonated molecule identified in this study. Fragmentation mechanisms of ferroverdins (compounds 1, 2, and 3) were obtained by HCD fragmentation of molecular ions (M-) 860.12, 876.11, and 904.11, respectively. Fragmentation mechanism data from bagremycins (compounds 4, 5, 6, 7, 8, and 9) were obtained by HCD fragmentation of molecular ions (M-H)^-^ 254.08, 269.09, 415.10, 239.07, 429.11, and 282.08, respectively. Download FIG S3, PDF file, 0.5 MB. Copyright © 2019 Martinet et al. 2019 Martinet et al. This content is distributed under the terms of the Creative Commons Attribution 4.0 International license.

### Bioinformatics.

The complete genome sequence of the S. lunaelactis MM109^T^ type strain was assembled from Illumina HiSeq and MiSeq short reads (Illumina, CA, USA) and Nanopore MinION long reads (Oxford Nanopore Technologies, United Kingdom) as described previously (28). Other S. lunaelactis strains genomes were assembled from Illumina reads only, using the complete genome sequence of the S. lunaelactis MM109^T^ type strain as the template (29).

The sequencing was performed at the GIGA-Research Center (Liège University, Belgium) (HiSeq and MinION) and at the Luxembourg Institute of Science and Technology (Belvaux, Luxembourg) (MiSeq).

Phylogeny analyses were performed with sequences of homolog proteins from the *fev* (S. lunaelactis MM109, *Streptomyces* sp. WK-5344), *bag* (*Streptomyces* sp. Tü 4128), *gri* (*S. griseus*), and *nsp* (S. murayamaensis) clusters. Proteins were aligned with MAFFT (v7.273, localpair mode), and alignments were trimmed and internal gaps removed. Phylogenetic inference was deduced using the maximum likelihood method as implemented in RAxML (v8.1.17, rapid bootstrapping mode with 1,000 replicates, GAMMA model of rate heterogeneity with WAG amino acid substitution matrix).
