# Supplementary figures and images for: A Single Biosynthetic Gene Cluster Is Responsible for the Production of Bagremycin Antibiotics and Ferroverdin Iron Chelators
Source: mBio. 2019 Aug 13;10(4):e01230-19. doi: 10.1128/mBio.01230-19 (PMC6692506; doi:10.1128/mBio.01230-19)

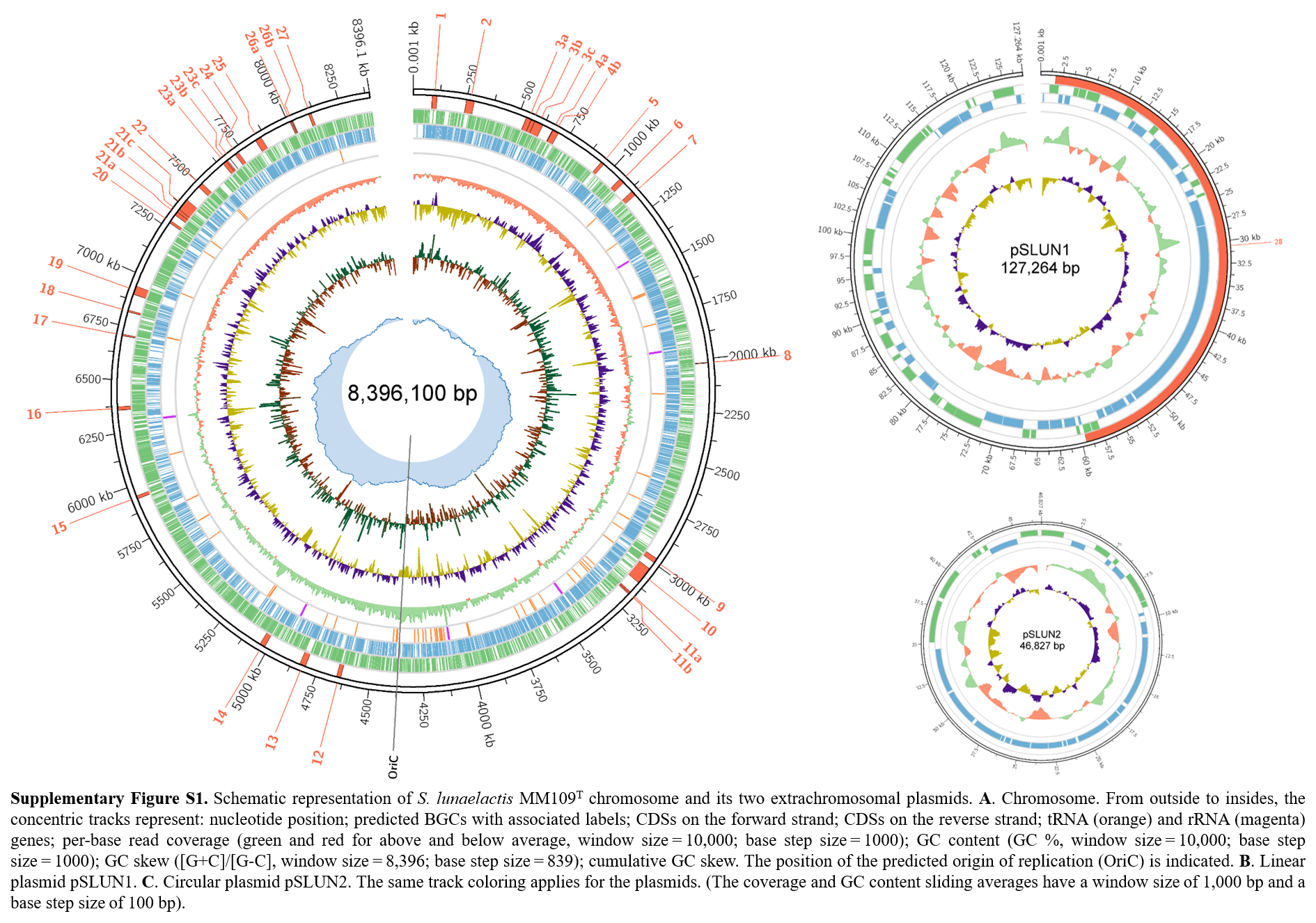

Supplement: FIG S1 [file mBio.01230-19-sf001.tif]

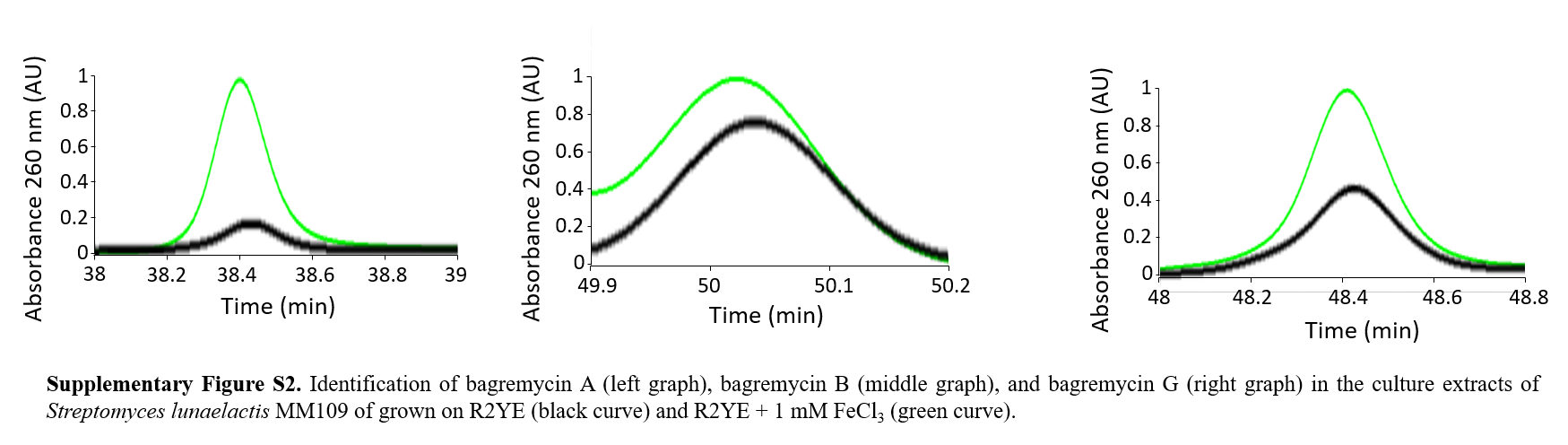

Supplement: FIG S2 [file mBio.01230-19-sf002.tif]
