# Supplementary material for: A Single Biosynthetic Gene Cluster Is Responsible for the Production of Bagremycin Antibiotics and Ferroverdin Iron Chelators
Source: mBio. 2019 Aug 13;10(4):e01230-19. doi: 10.1128/mBio.01230-19 (PMC6692506; doi:10.1128/mBio.01230-19)

Supplementary Figure S3. ESI(neg)–MS/MS spectra and proposed fragmentation mechanisms for each deprotonated molecule identified in this study. Fragmentation mechanisms of ferroverdins (compounds 1 to 3) obtained by HCD fragmentation of the molecular ions (M-) 860.12, 876.11 and 904.11 respectively. Fragmentation mechanisms of bagremycins (compounds 4 to 9) were obtained by HCD fragmentation of the molecular ions (M-H)- 254.08, 269.09, 415.10, 239.07, 429.11, and 282.08, respectively.

① **Ferroverdin A,** HRESIMS m/z 860.1199 [M – H]⁻ (calcd for $C_{45}H_{30}N_3O_{12}Fe$, 860.1173, Δm = 1.7 ppm)

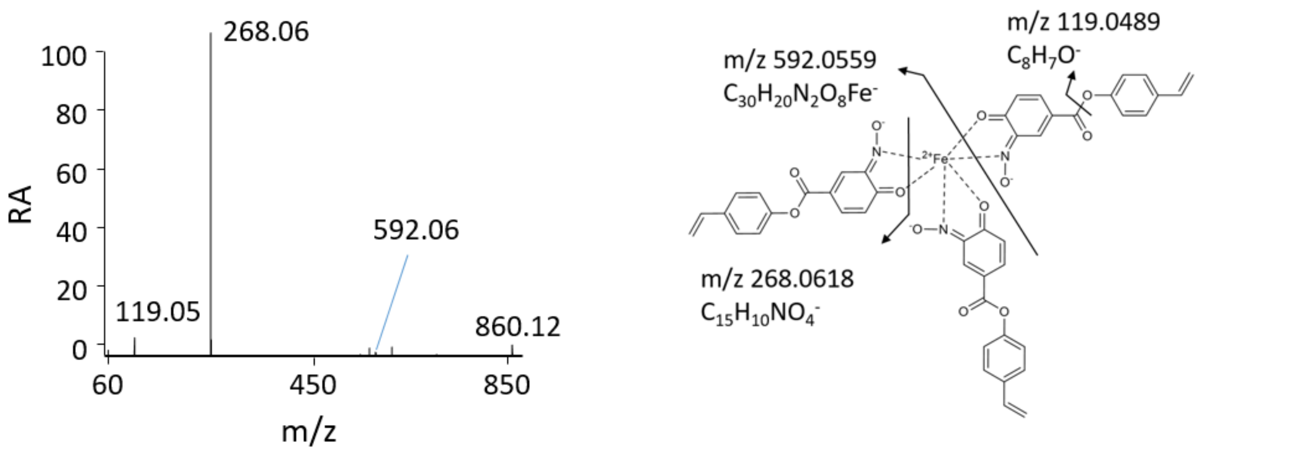

② **Ferroverdin B,** HRESIMS m/z 876.1149 [M – H]⁻ (calcd for $C_{45}H_{30}N_3O_{13}Fe$, 876.1123, Δm = 1.8 ppm)

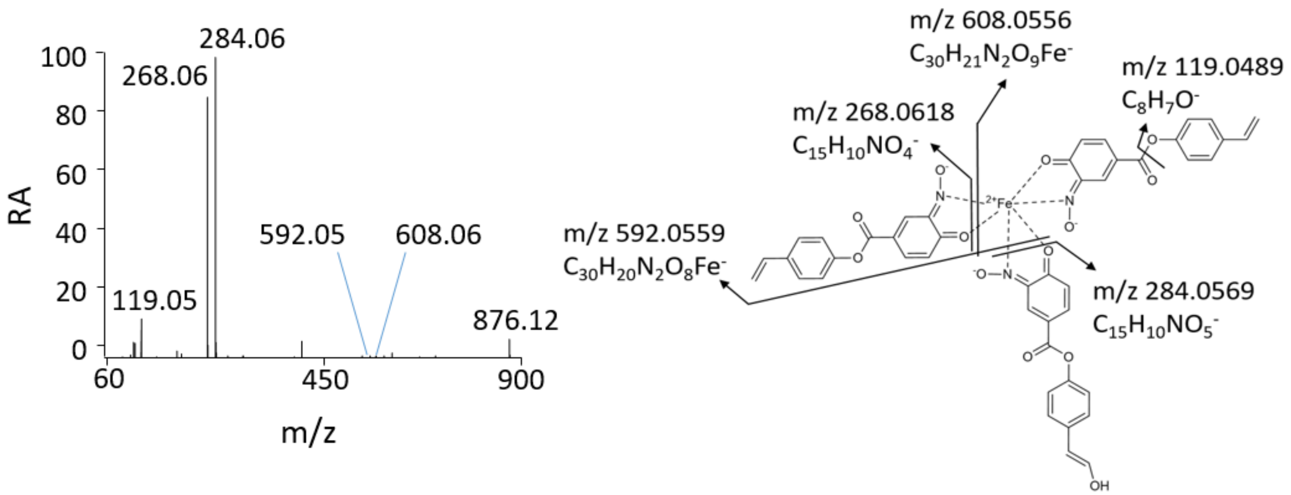

③ **Ferroverdin C,** HRESIMS m/z 904.1099 [M – H]⁻ (calcd for $C_{46}H_{30}N_3O_{14}Fe$, 904.1072, , Δm = 1.8 ppm)

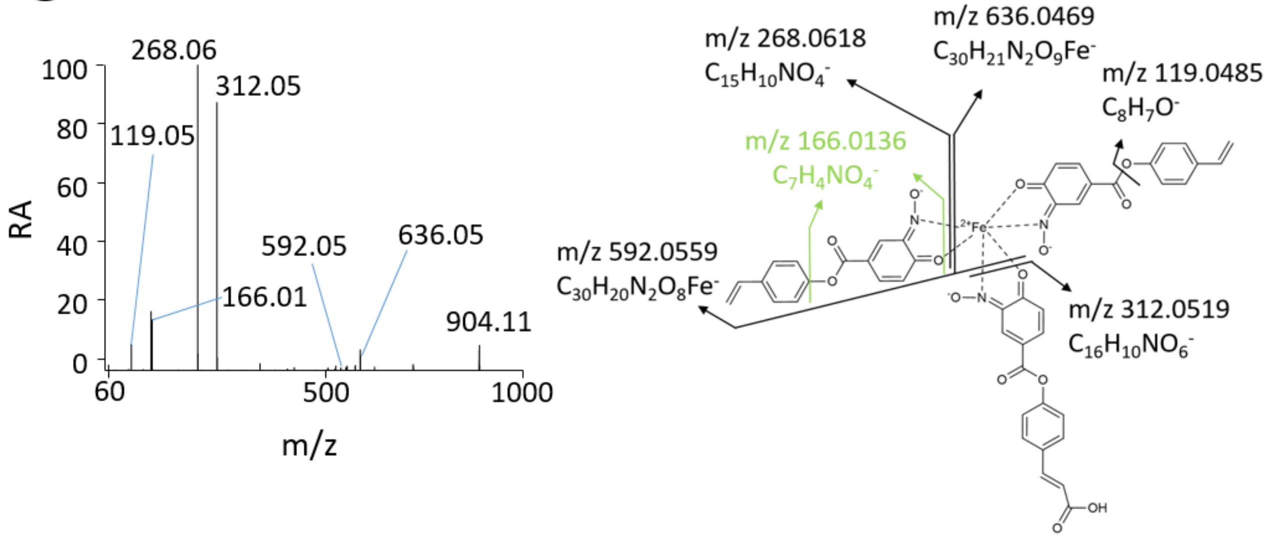

④ **Bagremycin A,** HRESIMS m/z 254.0824 $[M-H]^-$ (calcd for $C_{15}H_{12}NO_3$, 254.0812, Δm = 1 ppm) and $[M+H]^+$ 256.0967 (calcd for $C_{15}H_{14}NO_3$, 256.0968, Δm = 0.3 ppm)

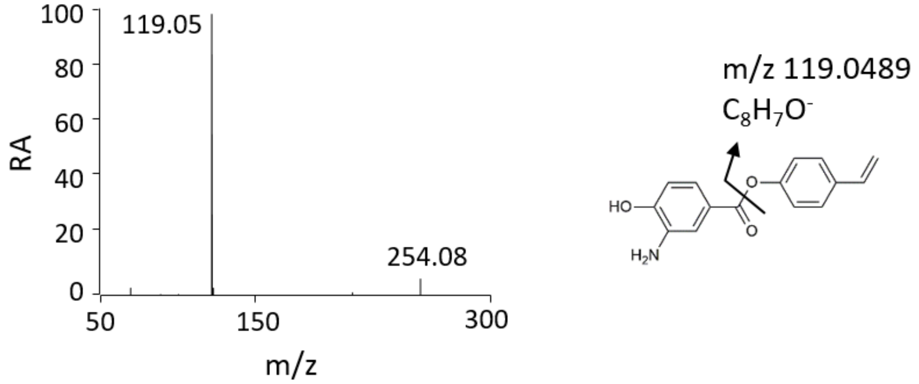

⑤ **Bagremycin B,** HRESIMS m/z 296.0931 $[M-H]^-$ (calcd for $C_{17}H_{14}NO_4$, 296.0917, Δm = 1.5 ppm) and $[M+H]^+$ 298.1073 (calcd for $C_{17}H_{16}NO_4$, 298.1074, Δm = 0.1 ppm)

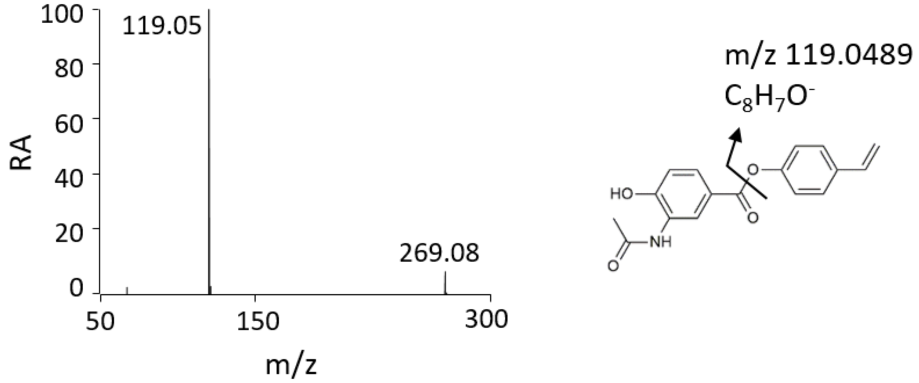

⑥ **Bagremycin C,** HRESIMS m/z 415.0974 $[M-H]^-$ (calcd for $C_{20}H_{19}N_2O_6S$, 415.0958, Δm = 2 ppm) and $[M+H]^+$ 417.1115 (calcd for $C_{20}H_{21}N_2O_6S$, 417.1115, Δm = 0.3 ppm)

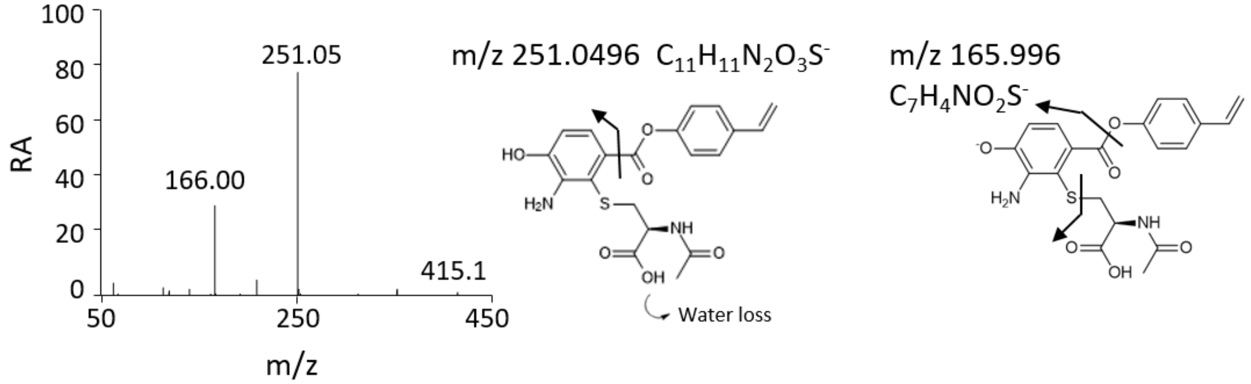

⑦ **Bagremycin E,** HRESIMS m/z 239.0714 $[M-H]^-$ (calcd for $C_{15}H_{10}O_3$, 239.0703, , Δm = 0.3 ppm) and $[M+H]^+$ 241.0859 (calcd for $C_{15}H_{12}NO_3$, 241.0859, , Δm = 0.3 ppm)

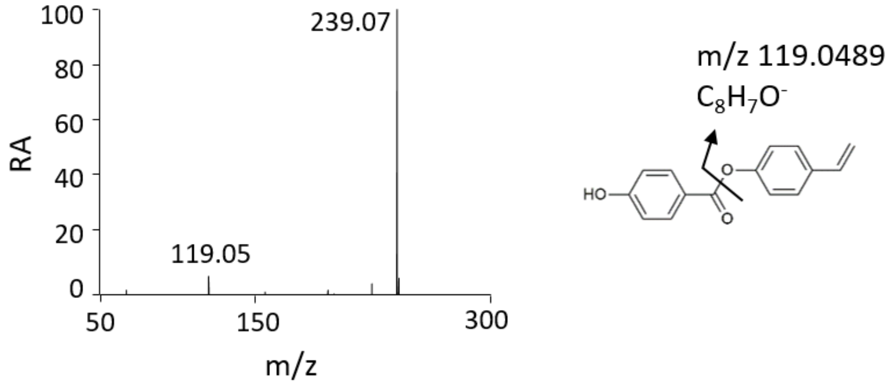

⑧ **Bagremycin F,** HRESIMS m/z 282.0777 [M – H]⁻ (calcd for $C_{16}H_{12}NO_4$, 282.0761, Δm = 1.9 ppm)

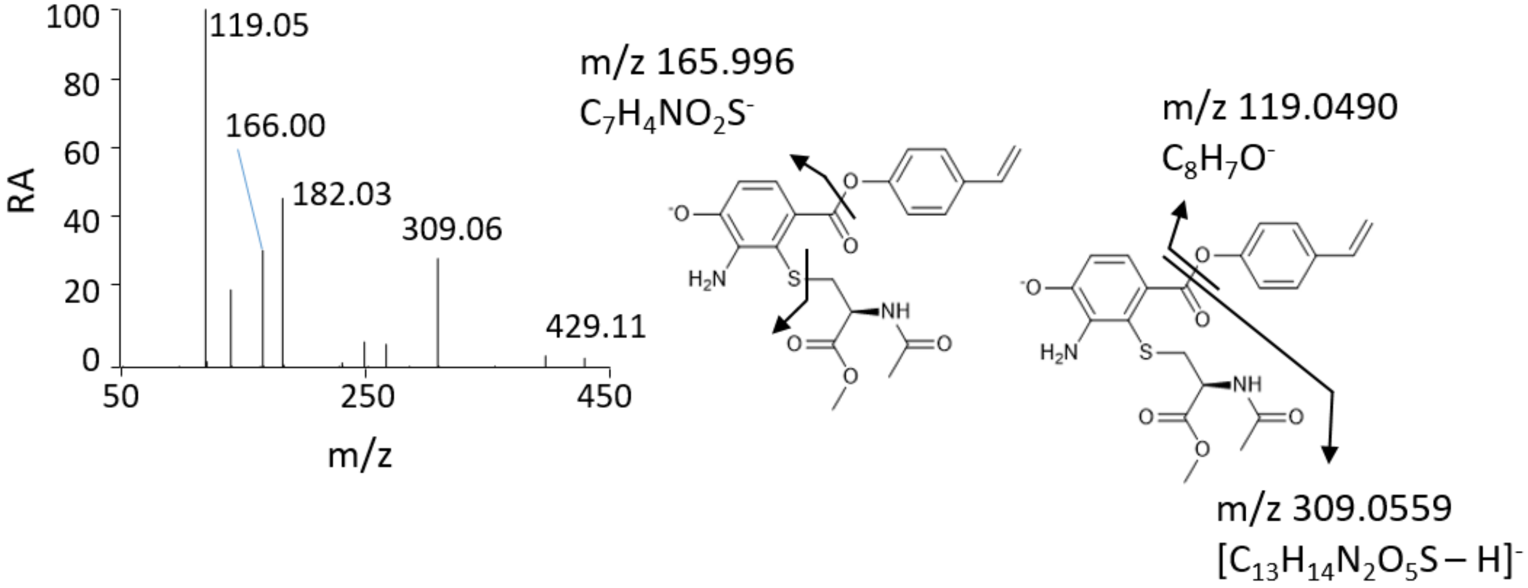

⑨ **Bagremycin G,** HRESIMS m/z 429.1133 [M – H]⁻ (calcd for $C_{21}H_{21}N_2O_6S$, 429.1115, Δm = 1.6 ppm)

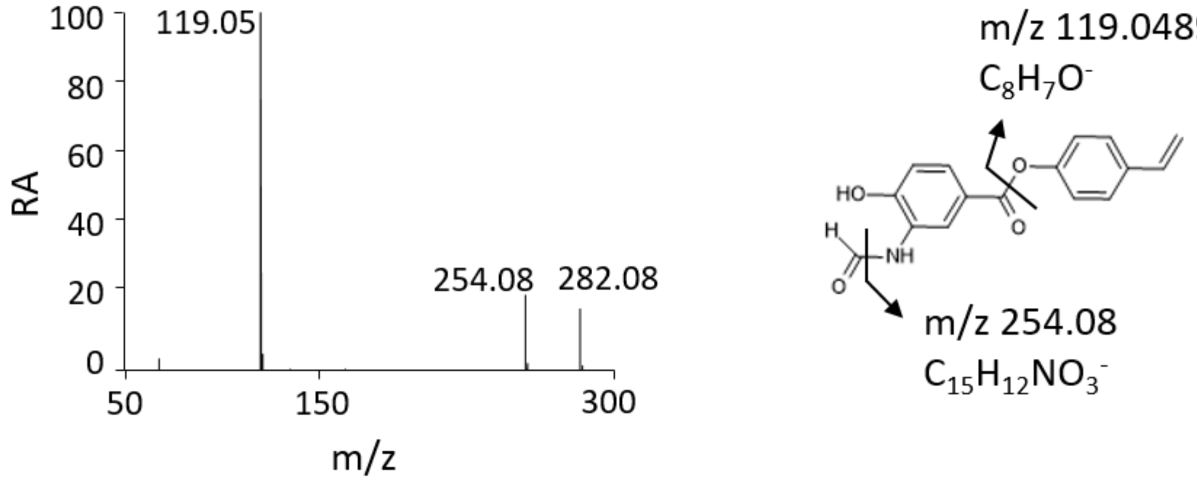

Supplement: FIG S3 [file mBio.01230-19-sf003.pdf]
